# Evaluation of the radioactive contamination in fungi genus *Boletus* in the region of Europe and Yunnan Province in China

**DOI:** 10.1007/s00253-015-6668-0

**Published:** 2015-06-06

**Authors:** Jerzy Falandysz, Tamara Zalewska, Grażyna Krasińska, Anna Apanel, Yuanzhong Wang, Sviatlana Pankavec

**Affiliations:** Gdańsk University, 63 Wita Stwosza Str., PL 80-803 Gdańsk, Poland; Institute of Meteorology and Water Management, National Research Institute, Maritime Branch Waszyngtona 42, PL 81-342 Gdynia, Poland; Institute of Medicinal Plants, Yunnan Academy of Agricultural Sciences, 2238 Beijing Road, Panlong District, Kunming, 650200 China

**Keywords:** China/Europe, Forest, Organic food, Radiocesium, Wild mushrooms

## Abstract

Numerous species of wild-grown mushrooms are among the most vulnerable organisms for contamination with radiocesium released from a radioactive fallout. A comparison was made on radiocesium as well as the natural gamma ray-emitting radionuclide (^40^K) activity concentrations in the fruiting bodies of several valued edible *Boletus* mushrooms collected from the region of Europe and Yunnan Province in China. Data available for the first time for *Boletus edulis* collected in Yunnan, China, showed a very weak contamination with ^137^Cs. Radiocesium concentration activity of *B. edulis* samples that were collected between 2011 and 2014 in Yunnan ranged from 5.2 ± 1.7 to 10 ± 1 Bq kg^−1^ dry matter for caps and from 4.7 ± 1.3 to 5.5 ± 1.0 Bq kg^−1^ dry matter for stipes. The mushrooms *Boletus badius*, *B. edulis*, *Boletus impolitus*, *Boletus luridus*, *Boletus pinophilus*, and *Boletus reticulatus* collected from the European locations between 1995 and 2010 showed two to four orders of magnitude greater radioactivity from ^137^Cs compared to *B. edulis* from Yunnan. The nuclide ^40^K in *B. badius* was equally distributed between the caps and stipes, while for *B. edulis*, *B. impolitus*, *B. luridus*, *B. pinophilus*, and *B. reticulatus*, the caps were richer, and for each mushroom, activity concentration seemed to be more or less species-specific.

## Introduction

The nuclear accident in Chernobyl, which took place on 26th of April 1986 caused large- scale diffusion of radioactivity mostly in the Central and Northern Europe, but it was detected also in other southern areas in Turkey (IAEA 2005; Simsek et al. 2014). Because of that accident, the long-term residual radioactivity in the affected areas comes largely from radiocesium (^137^Cs) (Bulko et al. 2014). Contamination of soils, pastures, and forests with the post-Chernobyl ^137^Cs varied between the regions of Europe, and this fact highly impacted on regional appearance of ^137^Cs in food, feed, mushrooms, grazing cattle, and wildlife and health risk of ^137^Cs and other nuclides to human consumers (Barret et al. 1999; Battiston et al. 1989; De Cort et al. 1998; Smith et al. 1993; Strandberg and Knudsen 1994; Zarubina 2014). Some but minor (about 10 %) residual radioactivity from ^137^Cs in the soils and wild-grown mushrooms still comes from the radioactive fallout which had taken place in the 1950s and 1960s because of the nuclear weapon tests in the atmosphere (Haselwandter et al. 1988; Steinhauser et al. 2013; Taira et al. 2011).

The nuclear power station in Fukushima Dai-ichi collapsed between 11th and 14th of March 2011 after a mega tsunami episode in the northeastern part of the Honshu Island—Tohoku region in Fukushima prefecture, Japan (Yasunari et al. 2011). In result of the Fukushima accident, a large-scale diffusion of radioactivity took place. The radioactivity plume was largely dispersed in the ocean and in small portion on land there (Teramage et al. 2014). At the local scale, mushrooms in the prefecture of Fukushima have been identified as the most relevant source of radiocesium intake among vegetables, especially after the first year of the accident (Merz et al. 2015). Foraging of mushrooms bypass, wittingly or unwittingly, the governmental food measuring campaigns which leads to higher intake of radioactive cesium than when consumers bought their products in commercial shops (Hayano et al. 2013; Normile 2013).

The airborne ^137^Cs that is deposited on land is efficiently taken up and sequestered in fruiting bodies by many wild-grown mushrooms (Macromycetes), which differ in their species-specific capacity to sequester stable Cs as well as many other metallic, non-metallic, and metalloid elements in the flesh (Bakken and Olsen 1990; Barret et al. 1999; Battiston et al. 1989; Brzostowski et al. 2011; Byrne et al. 1979; Eckl et al. 1986; Drewnowska and Falandysz 2015; Falandysz et al. 1994, 2003a, b, 2007a, b, c, d; Gucia et al. 2012; Kirchner and Daillant 1998; Kojta et al. 2012; Vinichuk et al. 2011; Zhang et al. 2010). These chemical elements, depending on their physical and chemical forms, can further be available from soil solution and soil bedrock to fungal mycelia, and sometimes, they can be sequestered in fungal flesh (fruiting bodies) more or less in a dose-effect-related manner. Hence, an elevated content of many metallic elements, metalloids, and Se (nonmetal) can be found in fruiting bodies of exposed populations, while the effectiveness of uptake and sequestration is a function of many variables including biological features related to species of mushroom, mycorrhiza, and geochemical/environmental factors (Falandysz and Borovička 2013).

Edible wild-grown mushrooms are popular organic food and are even items of international trade, and *Boletus* spp. are especially popular in Europe (King Bolete, Bay Bolete, Pine Wood) and especially in Yunnan and several other provinces in China (Falandysz et al. 2011; Wang et al. 2014). This paper reports and compares data on the residual activity from ^137^Cs as well as natural radionuclide from ^40^K accumulated in certain *Boletus* mushrooms collected in Poland, Sweden, and Belarus and in Yunnan of China. The contamination of King Bolete (*Boletus edulis*) from Yunnan (a land of mushrooms) is reported internationally for the first time. A major source of the residual ^137^Cs for Poland without doubt is the Chernobyl accident (Mietelski et al. 2010), while for Yunnan, the likely sources include radioactive fallout from nuclear weapon tests in the 1950s and 1960s and possibly also because of the Chernobyl and Fukushima accidents.

## Materials and methods

The fruiting bodies of *Boletus badius*, *B. edulis*, *Boletus impolitus*, *Boletus luridus*, *Boletus pinophilus*, and *Boletus reticulatus* mushrooms were collected in 1995–2014 in Poland, Belarus, and Sweden in Europe and in Yunnan in China. The mushrooms were collected across of Poland from different locations: Kacze Łęgi, Dziemiany, Sobieszewo, Mojusz, Kępice, Wdzydze, Parchowo, Bory Tucholskie, Olsztynek, Puszcza Piska, Puszcza Notecka, Porażyn, Włoszowa in Świętokrzyskie land, Chochołowska Valley in Tatra Mountains, and Kłodzka Dale in Sudety Mountains. Mushrooms from Belarus were collected from two spatially distant locations: Staroje Janczyna—location in central part, administrative circuit of Minsk, Borysowski Region, and Wasilewiczy in Chojniki area of the Gomel Region. Samples of *B. edulis* were also collected from the town of Umeå and its outskirts in northern region of Sweden and from Davingiie and Yimen in the Prefecture of Yuxi in the Yunnan Province of China. Fresh fruiting bodies’ samples (separately caps and stipes) were sliced using a plastic knife, dried at 65 °C to constant weight, and further pulverized using ceramic mortar and kept in brand new sealed polyethylene bags that were packed into larger bags and kept under dry and clean condition in a laboratory room until analysis. Before determination of activity concentration of radionuclides, the individual mushroom samples were pooled (separately caps and stipes, from 6 to 34 individuals per pool) to obtain one large (many specimens) integrated sample representing each place and year (Table 1).

**Table 1:** ^137^Cs and ^40^K in *Boletus* spp. (Bq kg^−1^ dry matter; activity concentration ± an instrumental counting error)

Place and year of collection (number of specimens, *n*) in a pool	^137^Cs		^40^K	
	Whole fruit bodies		Whole fruit bodies	
	Caps	Stipes	Caps	Stipes
*Boletus badius* Pers.				
(1)^a^ Poland, Bory Tucholskie, 2000 (*n* = 19)	5105 ± 45	4611 ± 48	1293 ± 52	1083 ± 43
(2) Poland, Puszcza Notecka, Jesionna, 2008 (*n* = 32)	970 ± 8	687 ± 19	1060 ± 28	713 ± 52
(3) Poland, Porażyn, 2008 (*n* = 29)	45 ± 2		1240 ± 55	
(4) Belarus, Borysów, Staroje Janczyna, 2010 (*n* = 34)	1430 ± 18	1373 ± 9	818 ± 136	828 ± 105
(5) Belarus, Chojniki, Wasilewiczy, 2010 (*n* = 38)	20,758 ± 196	14,799 ± 123	1090 ± 175	WD
*Boletus edulis* Bull.				
(6) Sweden, Umeå, and outskirts, 1995 (*n* = 15)	1102 ± 15	904 ± 12	904 ± 98	668 ± 95
(7) Poland, Pomerania, Mojusz, 2007 (*n* = 11)	1358 ± 17		912 ± 126	
(8) Poland, Pomerania, Parchowo, 2010 (*n* = 15)	497 ± 9	265 ± 4	731 ± 107	319 ± 76
(9) Poland, Tatra Mountains^b^, 1999 (*n* = 12)	227 ± 5		762 ± 111	
(10) Poland, Sudety Mt’s, Kłodzka Dale, 2000 (*n* = 10)	5722 ± 5	3485 ± 3	903 ± 118	368 ± 90
(20) China, Yunnan, Yuxi, Yimen, 2011 (*n* = 12)	10 ± 1	5.0 ± 1.0	740 ± 86	360 ± 61
(20) China, Yunnan, Yuxi, Yimen, 2012 (*n* = 10)	5.4 ± 1.2	5.5 ± 1.0	810 ± 74	500 ± 65
(21) China, Yunnan, Yuxi, Dayingjie, 2013 (*n* = 15)	5.2 ± 1.7	4.9 ± 1.1	630 ± 140	470 ± 91
(21) China, Yunnan, Yuxi, Dayingjie, 2014 (*n* = 15)	7.9 ± 1.5	4.7 ± 1.3	<120	<140
*Boletus impolitus *Fr.				
(11) Poland, Warmia land, Olsztynek, 2003 (*n* = 15)	276 ± 6	150 ± 4	608 ± 106	936 ± 91
*Boletus luridus Soverby*				
(12) Poland, Sobieszewo, 2000 (*n* = 23)	3533 ± 36	1007 ± 17	1008 ± 126	309 ± 136
(13) Poland, Pomerania, Kępice, 2003 (*n* = 15)	245 ± 8	72 ± 4	WD	WD
(14) Poland, Świętokrzyskie land^c^, 2007 (*n* = 12)	188 ± 6	102 ± 3	468 ± 122	218 ± 82
*Boletus pinophilus* Pilát & Dermek				
(15) Poland, Wdzydze Landscape Park, 1998 (*n* = 14)	970 ± 18	631 ± 9	631 ± 154	358 ± 87
(16) Poland, Pomerania, Dziemiany, 2000 (*n* = 14)	810 ± 14	425 ± 9	686 ± 120	WD
(17) Poland, Puszcza Notecka, Jesionna, 2000 (*n* = 6)	872 ± 17	564 ± 8	1075 ± 116	465 ± 108
(18) Poland, Puszcza Piska, 2000 (*n* = 14)	1195 ± 13	431 ± 6	638 ± 95	221 ± 91
*Boletus reticulatus* Schaeff.				
(19) Poland, TLP, Kacze Łęgi, 2006 (*n* = 20)	1094 ± 15	498 ± 8	905 ± 122	698 ± 101
(4) Belarus, Borysów, Staroje Janczyna, 2010 (*n* = 18)	393 ± 5	363 ± 8	790 ± 79	715 ± 119
(5) Belarus, Chojniki, Wasilewiczy, 2010 (*n* = 15)	6614 ± 109	3482 ± 30	687 ± 100	405 ± 102

*WD* without data, *TLP* Trójmiejski Landscape Park

**Fig. 1:**
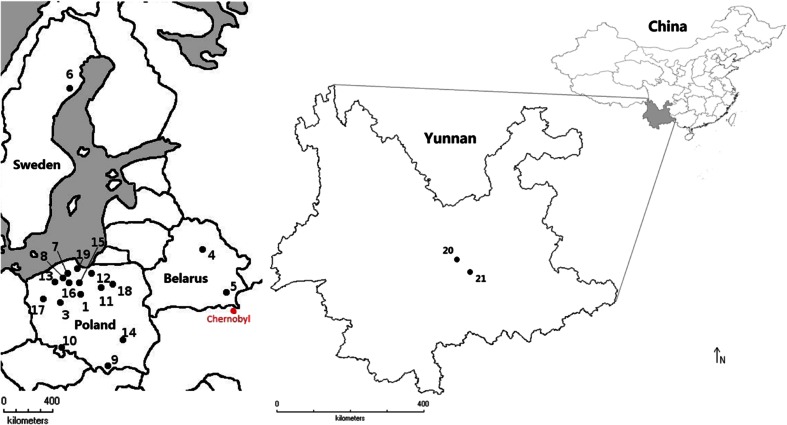
Localization of the sampling sites (*1*–*21*; for details see in Table 1)

^a^See at the map (Fig. 1)

^b^Chochołowska Valley

^c^Outskirts of the Włoszowa town

Activity concentrations of ^137^Cs and ^40^K were determined using gamma spectrometer with coaxial HPGe detector with a relative efficiency of 18 % and a resolution of 1.9 keV at 1.332 meV (with associated electronics). The detector was coupled with an 8192-channel computer analyzer and GENIE 2000 software (Zalewska and Staniewski 2011). The equipment was calibrated using a multi-isotope standard, and the method was fully validated. The laboratory involved was subjected for routine checks to ensure the high standards of analytical quality and analytical control as well as took part in the intercomparison exercises organized by IAEA-MEL Monaco (IAEA-414, Irish and North Sea Fish) (Zalewska and Staniewski 2011) to verify the reliability and accuracy of the method. All numerical data gained were recalculated for dehydrated fungal material (at 105 °C) and exact date of the sample collection.

## Results

Data on radioactivity (expressed in Bq kg^−1^ dry matter) of ^137^Cs and ^40^K in caps and stipes of the *Boletus* mushrooms are summarized according to species, place of origin, size of sample, and year of collection (Table 1). Samples of *B. edulis* were from Europe and China. The Chinese mushrooms were collected at altitude of 1600–1650 m above sea level in the Yuxi Prefecture of the mountainous Province of Yunnan in 2011–2014 (Fig. 1). The mushrooms such as *B. badius. B. edulis*, *B. impolitus*, *B. luridus*, *B. pinophilus*, and *B. reticulatus* collected in the region of Europe (Belarus, Poland, Sweden) in 1995–2010 showed two to four orders of magnitude (depending on species and place) greater activity concentration of ^137^Cs when compared to *B. edulis* collected in Yunnan in 2011–2014 (Table 1).

The nuclide, ^40^K, is a normal constituent of total K which is an important nutrient and the most abundant element in the fruiting bodies of mushrooms with a symbiotic or saprophyte life cycle. In *B. edulis* from Yimen in Yunnan, the activity of ^40^K was similar to that noted for the samples from Poland and Sweden (Table 1). Significantly lower values, less than 120 Bq kg^−1^ dm in caps and less than 140 Bq kg^−1^ dm in stipes, were found in mushrooms collected in 2014 in the Dayingjie region of Yunnan (the same was observed for ^40^K). This may be an indication of the deficiency of this important mineral nutrient in soils in Dayingjie, and this is worthy of further investigation.

Distribution of ^40^K between the two morphological parts of the fruiting bodies for *B. badius* (caps and stipes) was nearly equal for most of the sites. The exception to this pattern was for samples from Wasilewiczy (Table 1). For *B. edulis*, *B. luridus*, B. *pinophilus*, and *B. reticlatus*, the caps were frequently richer in ^40^K than the stipes, and only in the case of *B. impolitus* was the opposite characteristic observed.

Data obtained for ^137^Cs in *B. edulis* from Kłodzka Dale in the Sudety Mountains (southwestern Poland) showed relatively high contamination of samples with activity in caps of 5700 ± 2 Bq kg^−1^ dm. Also, samples of *B. edulis* from the region of Umeå in Sweden collected in 1995 (1102 ± 15 Bq kg^−1^ dm in caps and 904 ± 12 Bq kg^−1^ dm in stipes) and Mojusz in the Pomerania land of Poland collected in 2007 (1358 ± 17 Bq kg^−1^ dm in whole fruiting body) were substantially contaminated with ^137^Cs. The lowest activity concentrations of ^137^Cs in *B. edulis* were found in samples gathered in the other Pomeranian region (Parchowo) in 2010 (497 ± 9 Bq kg^−1^ dm in caps and 265 ± 4 Bq kg^−1^ dm in stipes) (Table 1). In contrast, the activity concentrations of ^137^Cs in *B. edulis* from the Yuxi region of Yunnan were very low, i.e., from 5.2 ± 1.7 to 10 ± 1 Bq kg^−1^ dm for caps and 4.7 ± 1.3 to 5.5 ± 1.0 Bq kg^−1^ dm for stipes (Table 1).

High activity of ^137^Cs were found also in other *Boletus* species: *B. luridus* collected from the forest with sandy soil bedrock at the Baltic Sea coastal place of the Sobieszewo Island near the city of Gdańsk, *B. pinophilus* from Puszcza Piska, and in *B. reticulatus* from Trójmiejski Landscape Park near the city of Gdynia (Table 1). The highest values were found in *B. luridus*; in 2000, the concentrations reached 3500 ± 36 Bq kg^−1^ dm in caps and 1000 ± 17 Bq kg^−1^ dm in stipes, while in *B. pinophilus* and in *B. reticulatus*, they were comparable and at the concentration of 1000 Bq kg^−1^ dm in caps and 400 Bq kg^−1^ dm in stipes. High levels of contamination with ^137^Cs were found in *B. reticulatus* from the outsktits of Wasilewiczy in the Chojniki Distict in the Gomel region of Belarus (Fig. 1). The activity found in this area was 6600 ± 109 Bq kg^−1^ dm in caps and 3500 ± 30 Bq kg^−1^ dm in stipes, lower than the values observed in *B. badius* collected at the same place and time, indicating possible differences in the ^137^Cs sequester capacity between these two species (Table 1).

## Discussion

*B. badius* is well known to be susceptible to contamination with radiocesium (Malinowska et al. 2006). The samples from the post-Chernobyl polluted region of Gomel in Wasilewiczy, Belarus, collected in 2010 contained high amounts of ^137^Cs, i.e., around 21,000 Bq kg^−1^ dry matter (dm) in caps and around 15,000 Bq kg^−1^ dm in stipes (Table 1). The activity of ^137^Cs in *B. badius* from Poland varied depending on the sampling locations, and this could be roughly related to the regional differences in deposition of ^137^Cs on and in soils because of the Chernobyl nuclear accident (Grodzinskaya and Haselwandter 2003; Haselwandter et al. 1988; Mietelski et al. 2010). The highest values of 5100 ± 45 Bq kg^−1^ dm in caps and 4600 ± 48 Bq kg^−1^ dm in stipes were found in samples from the Bory Tucholskie site collected in the year 2000. A slightly lower activity of ^137^Cs, 4800 ± 61 Bq kg^−1^ dm in caps and 2180 ± 190 Bq kg^−1^ dm in stipes, was found in *B. badius* collected in 1995–1996 from the complex forests of the Wdzydze Landscape Park which is very close to Bory Tucholskie (Malinowska et al. 2006; Falandysz et al. 2003a, b). The mushroom *B. badius* from two other large forest complexes of the Puszcza Notecka within the outskirts of Porażyn (Fig. 1) that was sampled in 2008 showed much lower levels of contamination when compared to the corresponding values for mushrooms sampled elsewhere in Poland in the 1990s by other researchers with 970 ± 8 Bq kg^−1^ dm in the caps and 45 ± 2 Bq kg^−1^ dm in the stipes (Table 1) (Malinowska et al. 2006).

The global radioactive fallout from nuclear weapon tests in the 1950s and 1960s and fallout from the Chernobyl accident has to be taken into account as a source of ^137^Cs accumulated in *B. edulis* growing in Europe (García et al. 2015; Malinowska et al. 2006; Mietelski et al. 2010). The ^137^Cs activity concentrations in *B. badius* and. *B. reticulatus* from Belarus, *B. badius* from Poland, and *B. edulis* from the Sudety Mountains in Poland are consistent with reported ^137^Cs general picture and “hot spot” deposition for regions of Belarus and Poland due to the Chernobyl accident (De Cort et al. 1998; Mietelski et al. 2010).

There is no information available to indicate that the most recent radioactivity release from the Fukushima accident affected Yunnan. Lack of gamma-ray radiation from ^134^Cs in mushrooms sampled in Yunnan directly after the Fukushima accident in 2011 up to 2014 in this study (Table 1) and two other reports indicated that the contribution of Fukushima to the total radiocesium deposited there should be considered as negligible (Falandysz et al. 2015; Wang et al. 2015). On the other hand, earlier (pre-Fukishima accident period) data on the occurrence of ^137^Cs in wild-grown mushrooms from Japan and Taiwan (in Asia) showed negligible contamination and thus can indirectly reflects depositions of small amounts of airborne ^137^Cs after the Chernobyl accident and previous nuclear weapon tests in the atmosphere (^134^Cs because of short life time, *t*_½_ = 2 years, decayed until Fukushima accident) (Muramatsu et al. 1991; Tsukada et al. 1998; Wang et al. 1998). Previous data on ^134^Cs and ^137^Cs in *Boletus* spp. from Yunnan and other regions of China are lacking. The activity concentrations from ^137^Cs for samples from Yimen and Dayingjie (Yuxi Prefecture) were of the same order of magnitude, and this may indicate similarities in radioactive fallout there.

Low activity concentrations of ^137^Cs determined in fruiting bodies of *B. edulis* from Yunnan presented in this study as well as in fruiting bodies of pan-tropical mushroom *Macrocybe gigantea* (median value for dehydrated caps was 4.5 Bq kg^−1^ and 5.4 Bq kg^−1^ for stipes) and sclerotia of fungus *Wolfiporia extensa* (range from <1.4 to 7.2 ± 1.1 Bq kg^−1^ dm) (Falandysz et al. 2015; Wang et al. 2015) definitely imply that radioactive contamination, which could have resulted from both the recent (Fukushima) and earlier (Chernobyl and/or nuclear weapon tests) sources, is negligible in this region.

The content of stable Cs in fruiting bodies of mushrooms such as *B. edulis* and *B. badius* and also *Cortinarius caperatus*, *Cortinarius saturatus*, *Cortinarius traganus*, *Dermocybe semisanguinea*, *Hydnum repandum*, *Laccaria amethystina*, *Lactarius allis*, *Lactarius piperatus*, *Lactarius rufus*, *Paxillus involutus*, *Suillus luteus*, *Tricholoma album*, *Tricholoma flavovirens*, *Tricholoma fulvum*, *Tricholoma robustum*, *Ramaria pallida*, *Sarcodon scabrum*, and *Xerocomus chrysenteron* was greater when compared to many others (Bakken and Olsen 1990; Byrne et al. 1979; Falandysz et al. 2001b, 2007a, b, c, 2008; Horyna and Řanda 1988; Karadeniz and Yaprak 2010; Tsukada et al. 1998; Yoshida et al. 2004). Nevertheless, data for ^134^Cs, ^137^Cs, and stable ^133^Cs obtained for the same samples of mushrooms available from published literature is little is little (Karadeniz and Yaprak 2010; Yoshida et al. 2000).

The relative abundance of ^137^Cs in mushroom as determined in this study for *Boletus* mushrooms from Europe can be attributed to three factors: species-specific uptake, requirement of this (stable Cs) element by the mushroom, and lastly by forest soil contamination with ^137^Cs at the sampling sites. Of secondary importance is the soil depth where the mushroom developed its mycelia, which is species-specific (Byrne 1998; Falandysz et al. 2014a; Stijve and Poretti 1990). The bulk of radioactive fallout as well as other airborne elemental contaminants are deposited and adsorbed on the top organic layer of forest soils. Some mushrooms with shallow mycelia can accumulate them readily and in considerable concentrations (Falandysz et al. 2014b; Mietelski et al. 2010; Stijve and Poretti 1990), and they subsequently infiltrate deeper into the soil layers (and the mycelia therein). This is dependent on the element’s concentration, while topography, humidity of climate, and soil structure can favor quicker vertical passage of the element under consideration into the deeper layers of the soil alongside with infiltrating rain, taking a portion of nuclides that were not readily adsorbed by litter and organic horizon of soil into deeper layers (Teramage et al. 2014).

The low concentrations of ^137^Cs found in *B. edulis* from Yunnan are important for the inhabitants of the region. One reason is because the *Boletus* mushrooms are very popular organic foods, and numerous species are collected in Yunnan, which is well known for the mushrooms that can be found there (Wang et al. 2014; Wiejak et al. 2014; Zhang et al. 2010). Another reason is that in Yunnan, *Boletes* mushrooms are traditionally fried with hot vegetable oil using a wok (Chinese pan) but without pre-boiling (blanching). Blanching is a common procedure when cooking or pickling *Boletus* mushrooms in many countries including Poland. It results in the leaching out of some of the mushrooms’ water and water-soluble constituents (including ^137^Cs) into the water phase, thereby reducing their content in the final mushroom dish (Barret et al. 1999).

The radioactive isotopes ^134^Cs and ^137^Cs can account for the “total” Cs (stable Cs) content when measured using an instrumental method that is unable to differentiate between ^134/137^Cs and ^133^Cs. This is particularly important because the content of ^134/137^Cs in mushrooms is associated with ^133^Cs (Karadeniz and Yaprak 2010; Yoshida et al. 2000).

As stated earlier, there is a dearth of data on ^137^Cs in mushrooms from China’s mainland (Marzano et al. 2001). No available information is found concerning the content of ^134^Cs and ^137^Cs, stable ^133^Cs, and ^40^K in *B. edulis* from Yunnan or other closely related species like *B. pinophilus or B. reticulatus*—all three are naturally rich in selenium and certain other chalcophile elements (Falandysz 2008, 2013; Falandysz et al. 2001a, 2007d, 2011; Frankowska et al. 2010; Costa-Silva et al. 2011). They and some other related species are naturally more abundant in stable ^133^Cs than many other mushrooms (Falandysz et al. 2001b, 2007d, 2008; Horyna and Řanda 1988).

The content of potassium (K) is high in fruiting bodies of the mycorrhizal type mushrooms, e.g., at 29,000 ± 3000 mg kg^−1^ dm in caps of *B. edulis*, from 38,000 ± 4000 to 55,000 ± 2000 mg kg^−1^ dm in *Cantharellus cibarius*, and from 28,000 ± 3000 to 50,000 ± 14,000 mg kg^−1^ dm in caps and from 21,000 ± 4000 to 35,000 ± 4000 in stipes of *Suillus grevillei* (Chudzyński and Falandysz 2008; Falandysz and Drewnowska 2015; Frankowska et al. 2010). Nuclide ^40^K is a long-living isotope and is a natural part of total K, which is an essential element and undergoes a homeostatic regulation in fruiting bodies by mushrooms (Falandysz and Borovička 2013; Stijve 1996).

^40^K is a dominant portion of the natural gamma-radioactivity contained in the flesh of the fruiting bodies of mushrooms (Karadeniz and Yaprak 2010). The activity concentration of ^40^K had a little fluctuation and was a substantial portion of the total gamma-radioactivity contained in the flesh of the fruiting bodies of all the *Boletus* mushrooms foraged in Europe in this study, and in practice, almost a solely source in samples from Yunnan, where >100-fold exceeded activity concentration of ^137^Cs.

In conclusion, the amount of ^40^K found in the fruiting bodies of a particular species of *Boletus* mushrooms collected from spatially distant places was more or less species-specific and stable with respect to time. On the other hand, a spatial pattern of activity of ^137^Cs in these mushrooms was mosaic-like, and this could be attributed to differences in the density of fallout and local soil conditions. For some areas of land located well away from the Chernobyl nuclear unit, the mosaic-like pattern of ^137^Cs accumulated in mushrooms (*Boletus* mushrooms) can reflect possible local differences in the density of fallout and the type of nuclides available to fungi when compared to what can be deduced from the expected pattern associated with pollution by ^137^Cs in European soils. To get a better knowledge on exposure rates and risk to consumers from ^137^Cs and other radionuclides in wild-grown mushrooms and especially the exposure to individuals, villagers and other high-level consumers of mushrooms need to be highlighted at any locality (e.g., forest) where mushrooms are highly contaminated.

## References

[CR1] Bakken LR, Olsen RA (1990). Accumulation of radiocaesium in fungi. Can J Microbiol.

[CR2] Barret CL, Beresford NA, Self PL, Howard BJ, Frankland JC, Fulker MJ, Dodd BA, Marriott IVR (1999). Radiocaesium activity concentrations in the fruit-bodies of macrofungi in Great Britain and an assessment of dietary intake habits. Sci Total Environ.

[CR3] Battiston GA, Degetto S, Gerbasi R, Sbrignadello G (1989). Radioactivity in mushrooms in Northeast Italy following the Chernobyl accident. J Environ Radioact.

[CR4] Brzostowski A, Falandysz J, Jarzyńska G, Zhang D (2011). Bioconcentration potential of metallic elements by poison Pax (*paxillus involutus*) mushroom. J Environ Sci Health Part A.

[CR5] Bulko NI, Shabaleva MA, Kozlov AK, Tolkacheva NV, Mashkov LA (2014). The ^137^Cs accumulation by forest-derived products in the Gomel Region. J Environ Radioact.

[CR6] Byrne AR (1998). Radioactivity in fungi in Slovenia, Yugoslavia, following the Chernobyl accident. J Environ Radioact.

[CR7] Byrne AR, Dermel M, Vaksel T (1979). Silver accumulation by fungi. Chemosphere.

[CR8] Chudzyński K, Falandysz J (2008). Multivariate analysis of elements content of larch bolete (*Suillus grevillei*) mushroom. Chemosphere.

[CR9] Costa-Silva F, Marques G, Matos CC, Barros AIRNA, Nunes FM (2011). Selenium contents of Portuguese commercial and wild edible mushrooms. Food Chem.

[CR10] De Cort M, Dubois G, Fridman SD, Germenchuk MG, Izrael YA, Janssens A, Jones AR, Kelly GN, Kvasnikova EV, Matvenko II, Nazarov IM, Pokumeiko YM, Sitak VA, Stukin ED, Tabuchny LY, Tsaturov YS, Avdyushin SI (1998) Atlas of caesium deposition on Europe after the Chernobyl accident. Luxembourg: Office for Official Publications of the European Communities 92-828-3140-X

[CR11] Drewnowska M, Falandysz J (2015). Investigation on mineral composition and accumulation by popular edible mushroom common chanterelle (*Cantharellus cibarius*). Ecotoxicol Environ Saf.

[CR12] Eckl P, Hofmann W, Türk R (1986). Uptake of natural and man-made radionuclides by lichens and mushrooms. Radiat Environ Biophys.

[CR13] Falandysz J (2008). Selenium in edible mushrooms. J Environ Sci Health C.

[CR14] Falandysz J (2013). Review: on published data and methods for selenium in mushrooms. Food Chem.

[CR15] Falandysz J, Borovička J (2013). Macro and trace mineral constituents and radionuclides in mushrooms: health benefits and risks. Appl Microbiol Biotechnol.

[CR16] Falandysz J, Drewnowska M (2015). Macro and trace elements in common chanterelle (*Cantharellus cibarius*) mushroom from the European background areas in Poland: composition, accumulation, dietary exposure and data review for species. J Environ Sci Health B.

[CR17] Falandysz J, Bona H, Danisiewicz D (1994). Silver content of wild-grown mushrooms from northern Poland. Z Lebensm Unters Forsch.

[CR18] Falandysz J, Gucia M, Frankowska A, Kawano M, Skwarzec B (2001). Total mercury in wild mushrooms and underlying soil substrate from the City of Umeå and its surroundings, Sweden. Bull Environ Contam Toxicol.

[CR19] Falandysz J, Szymczyk K, Ichihashi H, Bielawski L, Gucia M, Frankowska A, Yamasaki S (2001). ICP/MS and ICP/AES elemental analysis (38 elements) of edible wild mushrooms growing in Poland. Food Addit Contam.

[CR20] Falandysz J, Kawano M, Świeczkowski A, Brzostowski A, Dadej M (2003). Total mercury in wild-grown higher mushrooms and underlying soil from Wdzydze Landscape Park, Northern Poland. Food Chem.

[CR21] Falandysz J, Lipka K, Kawano M, Brzostowski A, Dadej M, Jędrusiak A, Puzyn T (2003). Mercury content and its bioconcentration factors at Łukta and Morąg, Northeastern Poland. J Agric Food Chem.

[CR22] Falandysz J, Kunito T, Kubota R, Brzostowski A, Mazur A, Falandysz JJ, Tanabe S (2007). Selected elements of poison Pax *Paxillus involutus*. J Environ Sci Health A.

[CR23] Falandysz J, Kunito T, Kubota R, Lipka K, Mazur A, Falandysz JJ, Tanabe S (2007b) Selected elements in Fly agaric *Amanita muscaria*. J Environ Sci Health A 42:1615–1623 10.1080/10934520701517853 17849303

[CR24] Falandysz J, Kunito T, Kubota R, Bielawski MA, Falandysz JJ, Tanabe S (2007). Selected elements in brown birch scaber stalk *Leccinum scabrum*. J Environ Sci Health A.

[CR25] Falandysz J, Frankowska A, Mazur A (2007). Mercury and its bioconcentration factors in king bolete (*Boletus edulis*) bull. Fr J Environ Sci Health A.

[CR26] Falandysz J, Kunito T, Kubota R, Bielawski L, Frankowska A, Falandysz JJ, Tanabe S (2008). Multivariate characterization of elements accumulated in king bolete *Boletus edulis* mushroom at lowland and high mountain regions. J Environ Sci Health A.

[CR27] Falandysz J, Frankowska A, Jarzyńska G, Dryżałowska A, Kojta AK, Zhang D (2011). Survey on composition and bioconcentration potential of 12 metallic elements in king bolete (*Boletus edulis*) mushroom that emerged at 11 spatially distant sites. J Environ Sci Health B.

[CR28] Falandysz J, Krasińska G, Pankavec S, Nnorom IC (2014). Mercury in certain *boletus* mushrooms from Poland and Belarus. J Environ Sci Health B.

[CR29] Falandysz J, Saba M, Dryżałowska A, Wang J, Zhang D (2014). Mercury in the fairy-ring of *Gymnopus erythropus* (Pers.) and *Marasmius dryophilus* (Bull.) P. Karst. mushrooms from the Gongga Mountain, Eastern Tibetan Plateau. Ecotoxicol Environ Saf.

[CR30] Falandysz J, Zhang J, Zalewska T, Apanel A, Wang Y, Wiejak A (2015). Distribution and possible dietary intake of radioactive ^137^Cs, ^40^K and ^226^Ra with the pantropical mushroom *Macrocybe gigantea* in SW China. J Environ Sci Health A.

[CR31] Frankowska A, Ziółkowska J, Bielawski L, Falandysz J (2010). Profile and bioconcentration of minerals by king bolete (*Boletes edulis*) from the płocka dale in Poland. Food Addit Contam B.

[CR32] García MA, Alonso J, Melgar MJ (2015). Radiocaesium activity concentrations in macrofungi from Galicia (NW Spain): influence of environmental and genetic factors. Ecotoxicol Environ Saf.

[CR33] Grodzinskaya B, Haselwandter W (2003). Radiocesium contamination of wild-growing medicinal mushrooms in Ukraine. Intern J Med Mushrooms.

[CR34] Gucia M, Kojta AK, Jarzyńska G, Rafał E, Roszak M, Osiej I, Falandysz J (2012). Multivariate analysis of mineral constituents of edible Parasol Mushroom (*Macrolepiota procera*) and soils beneath fruiting bodies collected from Northern Poland. Environ Sci Pollut Res.

[CR35] Haselwandter K, Berreck M, Brunner P (1988). Fungi as bioindicators of radiocaesium contamination: pre- and post-Chernobyl activities. Trans Br Mycol Soc.

[CR36] Hayano RS, Tsubokura M, Miyazaki M, Satou H, Sato K, Sakuma Y (2013). Internal radiocesium contamination of adults and children in Fukushima 7 to 20 months after the Fukushima NPP accident as measured by extensive whole-body-counter survey. Proc Jpn Acad Ser B.

[CR37] Horyna J, Řanda Z (1988). Uptake of radiocesium and alkali metals by mushrooms. J Radioanal Nucl Chem Lett.

[CR38] IAEA (2005) Worldwide Marine Radioactivity Studies—WOMARS, Radionuclides levels in Oceans and sea, IAEA-TECDOC-1429, ISBN 92-0-114904-2

[CR39] Karadeniz Ö, Yaprak G (2010). ^137^Cs, ^40^K, alkali-alkaline earth element and heavy metal concentrations in wild mushrooms from Turkey. J Radioanal Nucl Chem.

[CR40] Kirchner G, Daillant O (1998). Accumulation of ^210^Pb, ^226^Ra and radioactive cesium by fungi. Sci Total Environ.

[CR41] Kojta AK, Jarzyńska G, Falandysz J (2012). Mineral composition and heavy metals accumulation capacity of Bay Bolete’s (*Xerocomus badius*) fruiting bodies collected near a former gold and copper mining area. J Geochem Explor.

[CR42] Malinowska E, Szefer P, Bojanowski R (2006). Radionuclides content in *Xerocomus badius* and other commercial mushrooms from several regions of Poland. Food Chem.

[CR43] Marzano FN, Bracchi PG, Pizzetti P (2001). Radioactive and conventional pollutants accumulated by edible mushrooms (*Boletus* sp.) are useful indicators of species origin. Environ Res Sect A.

[CR44] Merz S, Shozugawa K, Steinhauser G (2015). Analysis of Japanese radionuclide monitoring data of food before and after the Fukushima nuclear accident. Environ Sci Technol.

[CR45] Mietelski JW, Dubchak S, Błażej S, Anielska T, Turnau K (2010). ^137^Cs and ^40^K in fruiting bodies of different fungal species collected in a single forest in southern Poland. J Environ Radioact.

[CR46] Muramatsu Y, Yoshida S, Sumiya W (1991). Concentrations of radiocesium and potassium in basidiomycetes collected in Japan. Sci Total Environ.

[CR47] Normile D (2013). Insistence on gathering real data confirms low radiation exposures. Science.

[CR48] Simsek V, Pozzoli L, Unal A, Kindap T, Karaca M (2014). Simulation of ^137^Cs transport and deposition after the Chernobyl nuclear power plant accident and radiological doses over the Anatolian Peninsula. Sci Total Environ.

[CR49] Smith ML, Taylor HW, Sharma HD (1993). Comparison of the post-Chernobyl ^137^Cs contamination of mushrooms from Eastern Europe, Sweden, and North America. Appl Environ Microbiol.

[CR50] Steinhauser G, Merz S, Hainz D, Sterba JH (2013). Artificial radioactivity in environmental media (air, rainwater, soil, vegetation) in Austria after the Fukushima nuclear accident. Environ Sci Pollut Res.

[CR51] Stijve T (1996). Potassium content and growth rate of higher fungi. Australoasian Mycol Newsl.

[CR52] Stijve T, Poretti M (1990). Radicesium levels in wild-growing mushrooms from various locations. Mushroom J Summer.

[CR53] Strandberg M, Knudsen H (1994). Mushroom spores and ^137^Cs in faeces of the roe deer. J Environ Radioact.

[CR54] Taira Y, Hayashidai N, Brahmanandhan GM, Nagayama Y, Yamashita SJ, Takahashi J, Gutenitc A, Kazlovsky A, Urazalin M, Takamura N (2011). Current concentration of artificial radionuclides and estimated radiation doses from ^137^Cs around the Chernobyl nuclear power plant, the Semipalatinsk nuclear testing site, and in Nagasaki. J Radiat Res.

[CR55] Teramage MT, Onda Y, Patin J, Kato H, Gomi T, Nam S (2014). Vertical distribution of radiocesium in coniferous forest soil after the Fukushima nuclear power plant accident. J Environ Radioact.

[CR56] Tsukada H, Shibata H, Sugiyama H (1998). Transfer of radiocaesium and stable caesium from substrata to mushrooms in a pine forest in Rokkasho-mura, Aomori, Japan. J Environ Radioact.

[CR57] Vinichuk M, Rosén K, Johanson KJ, Dahlberg A (2011) Cesium (^137^Cs and ^133^Cs), potassium and rubidium in macromycete fungi and sphagnum plants. *In* Radioisotopes - Applications in Physical Sciences. Singh N (ed.) pp. ISBN: 978-953-307-510-5. 496 pages, In Tech. doi:10.5772/22263

[CR58] Wang J-J, Wang C-J, Lai S-Y, Lin Y-M (1998). Radioactivity concentrations of ^137^Cs and ^40^K in Basidiomycetes collected in Taiwan. Appl Radiat Isot.

[CR59] Wang X-M, Zhang J, Wu L-H, Zhao Y-L, Li T, Li J-Q, Wang Y-Z, Liu H-G (2014). A mini-review of chemical composition and nutritional value of edible wild-grown mushroom from China. Food Chem.

[CR60] Wang Y-Z, Zalewska T, Apanel A, Zhang J, Zhang J, Maćkiewicz Z, Wiejak A, Falandysz J (2015) Artificial ^137^Cs and ^134^Cs and natural ^40^K in sclerotia of *Wolfiporia extensa* fungus collected across of the Yunnan land in China. J Environ Sci Health Part B 50. doi:10.1080/0361234.2015.1038958 10.1080/03601234.2015.1038958 26079339

[CR61] Wiejak A, Wang Y, Zhang J, Falandysz J (2014). Bioconcentration potential and contamination with mercury of pantropical mushroom *Macrocybe gigantea*. J Environ Sci Health B.

[CR62] Yasunari TJ, Stohl A, Hayano RS, Burkhart JF, Eckhart S, Yasunari T (2011). Cesium-137 deposition and contamination of Japanese soils due to the Fukushima nuclear accident. PNAS.

[CR63] Yoshida S, Muramatsu Y, Steiner M, Belli M, Pasquale A, Rafferty B, Rühm W, Rantavaara A, Linkov I, Dvornik A, Zhuchenko T (2000) Relationship between radiocesium and stable cesium in plants and mushrooms collected from forest ecosystems with different contamination levels. Proceedings of the 10th International Congress of the International Radiation Protection Association. May, Hiroshima P-11-244

[CR64] Yoshida T, Muramatsu Y, Dvornik AM, Zhuchenko TA, Linkov I (2004). Equilibrium of radiocesium with stable cesium within the biological cycle of contaminated forest ecosystems. J Environ Radioact.

[CR65] Zalewska T, Staniewski M (2011). Bioaccumulation of gamma emitting radionuclides in red algae from the Baltic Sea under laboratory conditions. Oceanologia.

[CR66] Zarubina NE (2014). Contamination of fungi with radionuclides after accident of Chernobyl NNP. Intern Nucl Saf J.

[CR67] Zhang D, Frankowska A, Jarzyńska G, Kojta AK, Drewnowska M, Wydmańska D, Bielawski L, Wang J, Falandysz J (2010). Metals of king bolete (*Boletus edulis*) collected at the same site over 2 years. Afr J Agric Res.

